# The Role of Hypoxia-Inducible Factor 1α in Determining the Properties of Castrate-Resistant Prostate Cancers

**DOI:** 10.1371/journal.pone.0054251

**Published:** 2013-01-16

**Authors:** Weranja K. B. Ranasinghe, Lin Xiao, Suzana Kovac, Mike Chang, Carine Michiels, Damien Bolton, Arthur Shulkes, Graham S. Baldwin, Oneel Patel

**Affiliations:** 1 Department of Surgery, The University of Melbourne, Austin Health, Heidelberg, Victoria, Australia; 2 Department of Urology, The University of Melbourne, Austin Health, Heidelberg, Victoria, Australia; 3 Laboratory of Biochemistry and Cellular Biology (URBC), NARILIS - NAmur Research Institute for LIfe Sciences - FUNDP-University of Namur, Namur, Belgium; Florida International University, United States of America

## Abstract

**Background:**

Castrate-resistant prostate cancer (CRPC) is a lethal condition in patients receiving androgen deprivation therapy for prostate cancer (PC). Despite numerous studies showing the expression of HIF1α protein under normoxia in PC cell lines, the role of this normoxic HIF1α expression in chemo-resistance and migration has not been investigated previously. As no method is currently available to determine which tumors will progress to CRPC, the role of HIF1α in PC and its potential for predicting the development of CRPC was also investigated.

**Methods:**

The effect of HIF1α protein knockdown on chemo-resistance and migration of PC3 cells was assessed by cell counting and Transwell assays, respectively. Translation efficiency of HIF1α mRNA was determined in PC cells using a HIF1α 5′UTR-luciferase construct. Clinical outcomes were correlated following the staining of 100 prostate tumors for HIF1α expression.

**Results:**

The CRPC-like cell lines (PC3 and DU145) expressed more HIF1α protein than an androgen sensitive cell line (LNCaP). Migration rate and chemo-resistance were higher in the PC3 cells and both were decreased when HIF1α expression was reduced. Increased translation of HIF1α mRNA may be responsible for HIF1α overexpression in PC3 cells. Patients whose tumors expressed HIF1α had significantly decreased metastasis-free survival and the patients who were on androgen-deprivation therapy had decreased CRPC-free survival on Kaplan-Meier analysis. On multivariate analysis HIF1α was an independent risk factor for progression to metastatic PC (Hazard ratio (HR) 9.8, p = 0.017) and development of CRPC (HR 10.0, p = 0.021) in patients on androgen-deprivation therapy. Notably the tumors which did not express HIF1α did not metastasize or develop CRPC.

**Conclusions:**

HIF1α is likely to contribute to metastasis and chemo-resistance of CRPC and targeted reduction of HIF1α may increase the responsiveness of CRPCs to chemotherapy. Expression of HIF1α may be a useful screening tool for development of CRPC.

## Introduction

Prostate cancer (PC) is the second most common cancer in men worldwide and continues to impose a significant disease burden and a growing worldwide healthcare problem. However, our understanding of the mechanisms that contribute to the development of PC is still limited [1]. Androgens and the androgen receptor (AR) are important regulators of stimulation and survival of prostate cancer cells. Androgen deprivation therapy (ADT) is the mainstay of treatment for metastatic and locally advanced prostate cancer. However, ADT eventually fails to maintain prostate cancer suppression in a majority of men with this condition.

Castrate-resistant prostate cancer (CRPC) is a lethal form of PC that may progress and metastasize rapidly. On development of CRPC, more than 84% of patients will have metastases [2]. Few biomarkers for prediction of CRPC have been described [3], [4], and currently there is no universal consensus on identifying which patients with PC will progress to CRPC. Furthermore the mechanisms resulting in the development and progression of CRPC remain poorly understood in part because of the limited availability of cell lines which closely model CRPC. The two widely used PC cell lines PC3 and DU145 are not considered as fully representative of CRPC cells since they were not isolated from prostate cancers that had relapsed after androgen deprivation therapy, and since they express little [5] if any AR [6], whereas AR is often over-expressed in CRPC tumors. However as PC3 and DU145 cells display some of the fundamental properties of a CRPC tumor including high migration (metastasis), androgen-independence and chemo-resistance similar to the CRPC cell line LNCaP C4-2 [7], and also share similar molecular properties, including depletion/mutation of mitochondrial DNA, which have been correlated with invasiveness and drug resistance [8], these two cell lines are frequently referred to as CRPC cells [9], [10], [11].

Hypoxia is a reduction in the normal concentration of tissue oxygen which occurs in many diseases including cancer. A hypoxic microenvironment within the prostate has been postulated to be responsible for the promotion of secondary genetic alterations and angiogenic stimulation, leading to a more aggressive cell phenotype and malignant progression [12]. The ability of cells to adapt to hypoxia is dependent on a set of hypoxia-inducible transcription factors (HIFs) which consist of a regulatory alpha (HIF1α) and a constitutive beta subunit (HIF1β). HIFs bind to the core sequence 5′-RCGTG-3′ in target promoters and induce more than 200 functionally diverse genes involved in cell survival [13]. The synthesis of HIF1α occurs via oxygen-independent mechanisms but its degradation is oxygen-dependent and involves prolyl hydroxylase, asparaginyl hydroxylase, the Von Hippel-Lindau protein and the proteasomal system [13].

Although HIF1α is over expressed in a number of human cancers [14], [15], the role of HIF1α in cancer progression is unclear. High concentrations of HIF1α in renal and breast cancer cell lines were shown to increase cancer cell survival, whereas in ovarian cancer high HIF concentrations contributed to increased apoptosis [13]. Dai and co-workers reported that acute hypoxia increased HIF1α expression and the motility and invasive capacity of three PC cell lines [16]. However, despite numerous studies showing the presence of HIF1α expression in normoxia, and a report that HIF1α signaling is upregulated in normoxic castration-resistant LNCaP C4-2 cells as compared to the parental LNCaP cells [17], the role of normoxic HIF1α expression is not well documented, and therefore formed the basis of our current study.

Similarly, despite high HIF1α expression in PC tissues [14], [18], [19], [20], [21], [22], [23], [24], its association with prognosis is inconclusive [25], [24], [26], [27]. However the consensus is that HIF1α is upregulated in prostate tumors [28], [24] and is a potent tumor-induced shield against oxidative stress or destruction by androgen deprivation, chemotherapy or radiation cytotoxicity [29]. Currently no studies have reported the relationships between HIF1α expression and the development of CRPC. Therefore, we aimed to investigate the role of HIF1α in the regulation of CRPC and to analyze its potential as a biomarker for prediction of the development of CRPC.

## Materials and Methods

### In Vitro Studies

#### Cell culture

The three human prostate cancer cell lines (PC3, DU145 and LNCaP) used in this study were generously donated by A/Prof. Ian Davis, Ludwig Institute for Cancer Research, Melbourne, and had been purchased from ATCC in 2009. Cell lines were cultured in RPMI 1640 medium (Invitrogen, Mulgrave, Australia) supplemented with 8% FBS and 100 U/mL penicillin. All cells were maintained at 37°C in a humidified incubator with 95% air and 5% CO_2_. The hypoxia-treated cells were cultured in the same way as the controls except that the gas phase contained 94% nitrogen (N_2_), 5% CO_2_ and 1% O_2_, with oxygen concentrations monitored and automatically adjusted by an electronic oxygen controller (ProOx Model 110, Biospherix, Redfield, NY).

#### Western blot analysis

Cells were washed once with ice-cold phosphate-buffered saline (PBS) and lysed with 0.1–0.2 ml pre-boiled sodium dodecyl sulphate (SDS) lysis buffer. Proteins were separated by SDS-polyacrylamide gel electrophoresis, and transferred onto a Hybond-C Extra nitrocellulose membrane (GE Healthcare, Rydalmere, Australia). HIF1α protein was detected with a monoclonal mouse anti-human HIF1α antibody (1∶1000, BD Biosciences, North Ryde, Australia) followed by a secondary goat anti-mouse horseradish peroxidase-conjugated antibody (1∶5000, Bio-Rad). As a loading control, blots were incubated with a horseradish peroxidase (HRP)-conjugated rabbit anti-GAPDH antibody (Santa Cruz Biotechnology, Santa Cruz, CA). Bands were visualized in a LAS 3000 Image Reader (Fujifilm, Brookvale, Australia), with an ECL Advance Western Blotting Detection Kit (GE Healthcare). Densitometric analysis of the protein bands was performed with MultiGauge software (Fujifilm).

#### Proliferation assay

For measurement of basal levels of proliferation, 2 × 10^5^ cells were cultured in a 6 well petri-dish in culture medium supplemented with FBS. Cells were washed with PBS at 24 hours and cultured for a further 48 hours in serum free medium. The cells which were to receive treatment were plated as above, and the media was removed at 24 hours and supplemented with FBS-free media before treatment with hydrogen peroxide (H_2_O_2_), cobalt chloride, 5-fluorouracil (5-FU) or hypoxia (1% O_2_) for 48 hours. Cells were counted using an automated cell counter (Countess®, Invitrogen).

### Migration/invasion Assays (Transwell Assay)

Prostate cancer cells were seeded at a density of 2 × 10^5^ cells in 250 µl per well of serum-free culture medium onto the upper chamber of polyethylene terephthalate filter membranes coated with fibronectin. The upper chambers were inserted into tissue-culture wells and 750 µl serum-free culture medium was added to the lower chamber. After incubation overnight at 37°C, non-migratory cells on the surface of the upper membrane were removed with a cotton swab, and cells that had migrated through the membrane pores and invaded the underside of the membrane were fixed with 90% methanol and stained with hematoxylin and eosin. For quantitative assessment, the number of stained, migrating cells was then counted under a microscope. Five low-power fields per filter were counted on three separate occasions for three independent experiments.

#### Stable HIF1α knock down in PC3 cells

Plasmids encoding human HIF1α shRNA (Mission® clone numbers TRCN0000003810 and TRCN0000010819, which encode a hairpin-type siRNA) and a negative control plasmid (SHC002) were purchased from Sigma-Aldrich (St. Louis, MO). Cells were transfected with either a HIF1α shRNA plasmid or the negative control plasmid using the Neon® transfection method (Invitrogen). Briefly, 1 × 10^6^ cells were trypsinized, washed with PBS, and pelleted before resuspension in 100 µl Neon resuspension buffer. HIF1α or control shRNA plasmid (5 µg) were added and mixed well into the cell suspension prior to transfection. The transfected cells were seeded in complete medium and selected with 1.0 µg/ml puromycin (Sigma-Aldrich) for 7 days before further assays. Knockdown of the HIF1α protein was demonstrated by Western blotting, and by measurement of its downstream product, vascular endothelial growth factor (VEGF), by enzyme-linked immunosorbent assay (ELISA).

#### Translation efficiency

To determine whether the 5′UTR of HIF1α mRNA has any role in HIF1α overexpression in prostate cells a reporter plasmid was constructed in which the entire 5′UTR and 238 bp of the HIF1α promoter sequence was cloned upstream of *Firefly* luciferase coding sequences in the pGL4.10 reporter plasmid. The reporter construct was transfected into LNCaP and PC3 cells and the firefly luciferase activity driven by the HIF1-UTR reporter vector and *Renilla* luciferase (pTK-Renilla control reporter vector) were determined following 24 hours of incubation. Total mRNA was extracted from the transfected cells and the quantity of luciferase mRNA was determined using Real Time PCR. Luciferase mRNA in the cells transfected with the empty pGL4 vector was used as the basal control. Translation efficiency was calculated by dividing the *Firefly* luciferase activity (proportional to luciferase protein) by the concentration of *Firefly* luciferase mRNA. This ratio was further corrected for transfection efficiency by taking into account the *Renilla* luciferase activity.

### Clinical Outcome Studies

#### Human tissue samples

For the assessment of associations between HIF1α expression and clinical outcomes, 100 human prostate tumors from patients who had provided informed written consent were collected following radical prostatectomy or transurethral resection of the prostate (TURP) at our institution between 2000 and 2011. All samples were obtained from the Victorian Cancer Biobank and or the Department of Anatomical Pathology at the Austin Hospital, Victoria, Australia. Approval for the use of biological specimens and de-identified patient data for this study was obtained from the Austin Health Human Research Ethics Committee.

#### Immunohistochemistry

Paraffin-embedded tissue sections were de-waxed in histolene and hydrated with decreasing ethanol concentrations. Slides were rinsed in Tris-buffered saline/Tween20 (TBST) and the antigens were retrieved by heating in citric acid buffer (pH 6.0) in a microwave for 2 minutes on medium high and 13 minutes on medium low. Slides were allowed to cool and endogenous peroxidases in the specimens were blocked by treatment with 3% hydrogen peroxide for 10 minutes in the dark. Slides were washed in water, equilibrated in TBST buffer, blocked with ultravision (Thermo Fisher Scientific) for 10 minutes and stained for HIF1α using a HIF1α polyclonal antibody (1∶100, Santa Cruz Biotechnology, Santa Cruz, CA) at 4°C overnight. After antibody incubation, slides were treated with a HRP-conjugated secondary antibody (Dako) in the dark at room temperature for 1 hour. Slides were washed with TBST following 5 minutes incubation with 3,3′-diaminobenzidine (DAB) chromogen (1 drop/ml of substrate buffer, Dako) to complete colour development. Finally, slides were counterstained with hematoxylin for 1 minute, washed in running water and Scott’s tap water for 1 minute each, dehydrated and cover slipped. The investigators were blinded to the status of individual samples, and tumors were divided according to the presence or absence of HIF1α rather than weak or strong staining to reduce the inter-observer variation.

#### Outcomes

Distant metastases were defined by abnormalities documented on bone-scan or computed tomography. CRPC was defined as 2 consecutive rises of prostate-specific antigen (PSA) from the PSA nadir. The time to development of distant metastasis was measured from surgery, while the time to development of CRPC, chemo-resistance and PC-specific death were measured from the start of androgen deprivation. Pre-interventional PSA was defined as PSA immediately prior to obtaining the tissue sample, and T3 and T4 staging was defined as locally advanced prostate cancer as in the 2002 American Joint Commission on Cancer staging system [30].

#### Statistical analysis

Statistical analysis was performed under the guidance of the statistical counseling service, University of Melbourne, Australia. Pearson’s Chi-squared analysis and Fisher’s exact tests were conducted using two-by-two tables (Gleason score, HIF1α positivity, tumor stage and number of patients started on androgen deprivation therapy) on SigmaStat software (Jandel Scientific, San Rafael, CA) to test the association between the patient characteristics and HIF1α expression. Univariate and multivariate analysis were performed using Cox regression models for all variables. In order to overcome the non-convergence of the Cox regression model when analyzing for HIF1α positivity and Gleason score (as there were no outcomes in the HIF1α negative group and the low Gleason scores), these univariate and multivariate analysis were calculated by Cox regression with Firth’s penalized maximum likelihood method using R software, (R foundation for Statistical Computing version 2.14.0). Survival was calculated for each outcome using Kaplan-Meier curves with log rank test on the SPSS statistical package (IBM SPSS version 17). Diagnostic test evaluations with sensitivity and specificity analysis were performed using MedCalc statistical software (MedCalc Software, Belgium http://www.medcalc.org/).

*In vitro* data are presented as means ± SEM. Statistical significance for single comparisons of normally distributed data was determined by Student’s t test or for data that was not normally distributed by Mann-Whitney rank sum test. For multiple comparisons one-way ANOVAs followed by the Bonferroni correction were performed. All statistics were analyzed with the program SigmaStat (Jandel Scientific).

## Results

### HIF1α Expression Correlates with Migration Rate in PC Cells

**Figure 1:**
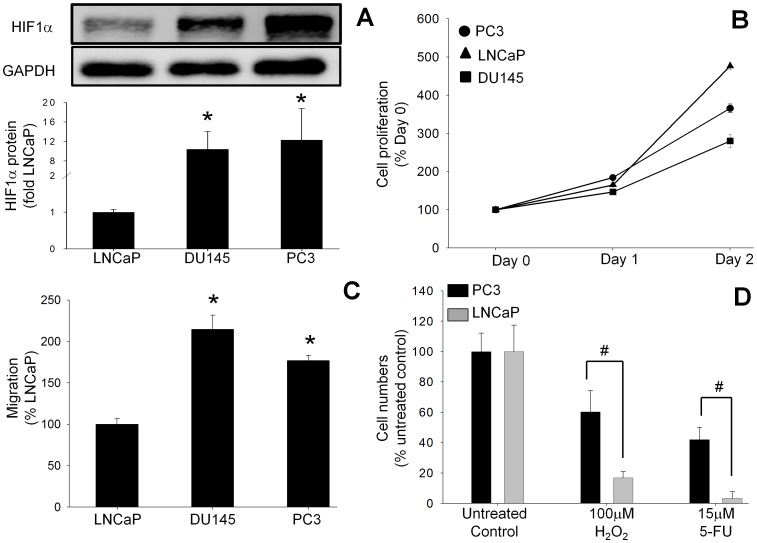
Basal HIF1α protein expression, proliferation rates and migration/invasion rates in human PC cell lines. ( A) Basal HIF1α protein concentrations in the human PC cell lines LNCaP, DU145 and PC3 under normoxic conditions were analyzed by Western blot. (B) Proliferation was assayed by cell counting after 24 and 48 hours. (C) Migration/invasion rates were measured by Transwell assays at 24 hours. Values in (A) and (C) are expressed as the fold increase compared to LNCaP cells, while the values in (B) are expressed as a percentage of the time 0 value. All values are the mean ± SEM of at least three separate treatments. (D) Survival rates of PC cells exposed to cytotoxic conditions. The survival of PC3 cells (which have higher basal HIF1α protein) when exposed to oxidative stress with hydrogen peroxide (H_2_O_2_) or chemotoxicity with 5-fluorouracil (5-FU) was compared to the survival of LNCaP cells (which have lower HIF1α expression). Survival was assessed by counting cell numbers at 24 hours. Values are expressed as a percentage of the untreated control and are the mean ± SEM of at least three separate treatments. #, P<0.05 versus treated LNCaP cells.

HIF1α protein expression was analyzed in androgen-sensitive (LNCaP) and androgen-insensitive (PC3 and DU145) CRPC-like cells. Basal HIF1α expression under normoxia was higher by 12±6-fold and 10±4-fold respectively in the CRPC-like cell lines PC3 and DU145 as compared to androgen-sensitive LNCaP cells (Figure 1A). Interestingly the observation that the basal growth rate of LNCaP cells in serum-free conditions was much higher than PC3 cells, which express more HIF1α, indicates that increased expression of HIF1α does not necessarily lead to greater proliferation (Figure 1B). To investigate the metastatic potential of the PC cell lines, migration of the CRPC cell lines PC3 and DU145 was compared to androgen-sensitive LNCaP cells using a Transwell assay. The observation that migration of PC3 and DU145 cells was 177±6% and 215±17% respectively of the value for LNCaP cells (100%) (Figure 1C) indicated that overexpression of HIF1α is associated with increased migration.

### Greater HIF1α Expression Correlates with Increased Cell Survival

One of the characteristics of CRPC is its resistance to chemo- and radio-therapy. To investigate whether HIF1α is responsible for the increased survival of CRPC-like cells *in vitro*, cell survival following treatment of PC3 or LNCaP cells with two cytotoxic agents, H_2_O_2_ (a source of oxidative stress) and 5-fluorouracil (5-FU, a chemotherapeutic drug) was measured. Cell proliferation assays (Figure 1D) revealed that the survival rates of 60±14% and 42±8% for PC3 cells following treatment with 100 µM H_2_O_2_ or 15 µM 5-FU respectively, were significantly greater than the respective survival rates of 17±4% and 3±1.6% in androgen-sensitive LNCaP cells.

### HIF1α Knockdown Decreases Cell Survival and Migration of PC3 Cells

**Figure 2:**
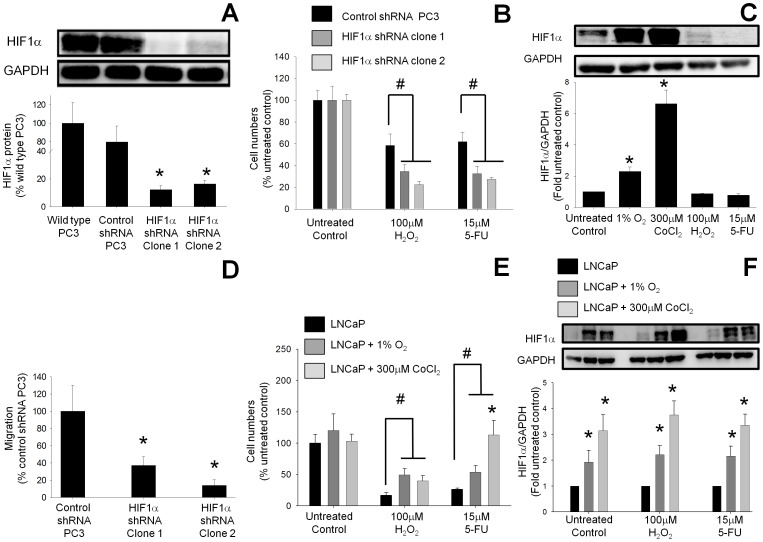
Knockdown of HIF1α expression in PC3 cells reduced both survival after cytotoxic treatments and migration rate. (A) HIF1α concentrations were reduced in 2 separate clones of PC3 cells following stable expression of HIF1α shRNA as assessed by Western blot. Values are the mean ± SEM of at least three separate experiments and are expressed as a percentage of wild-type PC3 cells. *, P<0.05 versus wild-type PC3 cells. (B) The survival of PC3 cells after exposure to oxidative stress (hydrogen peroxide (H_2_O_2_)) or chemotoxicity (5-fluorouracil (5-FU) for 24 hours was reduced following HIF1α knockdown compared to scrambled control vector-transfected PC3 cells. Values are the mean ± SEM of at least three separate experiments and are expressed as a percentage of untreated scrambled control vector-transfected PC3 cells. #, P<0.05 versus control. (C) HIF1α protein expression in PC3 cells transfected with control shRNA after treatment with 1% O_2_, 300 µM CoCl_2_, 100 µM H_2_O_2_, and 15 µM 5-FU. Cell lysates were electrophoresed on SDS-polyacrylamide gels and blotted with HIF1α antibody. GAPDH expression was used as loading control. The Western blots shown are representative of at least three separate experiments. Band densities were determined by densitometric analysis of HIF1α/GAPDH and are presented relative to the value for untreated cells. Data represent mean ± SEM; * p<0.05 vs. untreated PC3 cells. (D) Rates of migration/invasion in the HIF1α knockdown PC3 cells were reduced compared to the scrambled control vector-transfected PC3 cells as assessed by Transwell assay. Values are the mean ± SEM of at least three separate experiments and are expressed as a percentage of untreated scrambled control vector transfected PC3 cells. *, P<0.05 versus control. (E) Induction of HIF1α in LNCaP cells by hypoxia (dark grey bars) or by cobalt chloride (light grey bars) increased survival after exposure to oxidative stress with H_2_O_2_ or chemotoxicity with 5-FU for 24 hours when compared to control LNCaP cells (black bars). Values are the mean ± SEM of at least three separate treatments and are expressed as a percentage of the untreated LNCaP control. #, P<0.05 versus treated LNCaP cells. *, P<0.05 versus LNCaP cells treated with 1% O_2_ and 5-FU. (F) HIF1α protein expression in LNCaP cells treated with 1% O_2_ and 300 µM CoCl_2_ in combination with either 100 µM H_2_O_2_ or 15 µM 5-FU. Cell lysates were electrophoresed on SDS-polyacrylamide gels and blotted with HIF1α antibody. GAPDH expression was used as loading control. The Western blots shown are representative of at least three separate experiments. Band densities were determined by densitometric analysis of HIF1α/GAPDH and are presented relative to the value for normoxic cells undergoing the same treatment. Data represent mean ± SEM; * p<0.05 vs. untreated control, 100 µM H_2_O_2_ or 15 µM 5-FU treated LNCaP cells.

To confirm that the increased survival and migration of PC3 cells was due to greater HIF1α protein expression, the expression of HIF1α in PC3 cells was knocked down using shRNA vectors. Transfection of PC3 cells with a vector expressing HIF1α shRNA reduced the HIF1α protein expression to 12±3% in clone 1 and 16±3% in clone 2 compared to wild type PC3 cells (100%) (Figure 2A). VEGF, a downstream product of HIF1α, was also decreased in the HIF1α knockdown clones, confirming the reduction in HIF1α activity (data not shown). The observation that there was no difference in the basal proliferation rate between HIF1α shRNA-expressing PC3 cells and PC3 cells transfected with a scrambled control vector is consistent with the finding that there was no correlation between greater HIF1α expression and proliferation rate (Figure 1B). Following H_2_O_2_ treatment only 22.5±3% of the HIF1α knockdown PC3 cells (shRNA clone 1) survived as compared to 58.5±10% survival in scrambled control vector-transfected PC3 cells (Figure 2B). Similarly, 5-FU reduced the cell survival to 27±2% in the HIF1α knockdown cells, compared to 62±9% cell survival in PC3 cells transfected with a scrambled control vector. There was no significant change in the expression of HIF1α following the treatment of PC3 cells transfected with control shRNA with 100 µM H_2_O_2_ or 15 µM 5-FU as compared to untreated PC3 cells (Figure 2C). However there were 2.3±0.2-fold and 6.6±1-fold increases in the expression of HIF1α following the treatment of PC3 cells with 1% O_2_ or 300 µM CoCl_2_, respectively (Figure 2C). As shown in Figure 2A the basal expression of HIF1α in HIF1α shRNA expressing PC3 cells is undetectable, and therefore it is not feasible to determine the effect of treatment with 100 µM H_2_O_2_ or 15 µM 5-FU on HIF1α shRNA-expressing PC3 clones.

Previously a 1.8-fold greater migration rate was observed in PC3 cells compared to androgen-sensitive LNCaP cells. To determine whether or not this difference was mediated by HIF1α, the migration of HIF1α knockdown and control vector transfected-PC3 cells was compared. Knockdown of HIF1α expression by RNA interference decreased PC3 migration to 37±10% (clone 1) and 14±7% (clone 2), compared to the control vector transfected-PC3 cells (100%) (Figure 2D).

### Induction of HIF1α in LNCaP Cells Increases Cell Survival

To determine whether induction of HIF1α expression in androgen-sensitive LNCaP cells can increase their survival following treatment with cytotoxic agents, HIF1α expression was induced using either hypoxia (1% O_2_) or the hypoxia mimetic cobalt chloride (Figure 2E). Incubation of LNCaP cells with either 100 µM H_2_O_2_ or 15 µM 5-FU reduced the survival to 17±4% and 26±2% respectively compared to untreated control (100%). However the survival rates in the presence of 100 µM H_2_O_2_ following the treatment of LNCaP cells with either 1% O_2_ or cobalt chloride increased to 40±8% and 49±11% respectively. The survival (113±23%) of LNCaP cells treated with 300 µM CoCl_2_ in combination with 15 µM 5-FU was significantly higher compared to the survival (54±10%) observed in LNCaP cells treated with 1% O_2_ in combination with 15 µM 5-FU. Interestingly, 300 µM CoCl_2_ in combination with 15 µM 5-FU induced a slightly higher 3.3±0.5-fold increase in HIF1α expression in LNCaP cells as compared to the 2.1±0.4-fold increase induced by 1% O_2_ in combination with 15 µM 5-FU, although the difference was not statistically significant (Figure 2F).

### Increased Translation Efficiency of HIF1α mRNA is Responsible for HIF1α Overexpression in PC3 Cells

**Figure 3:**
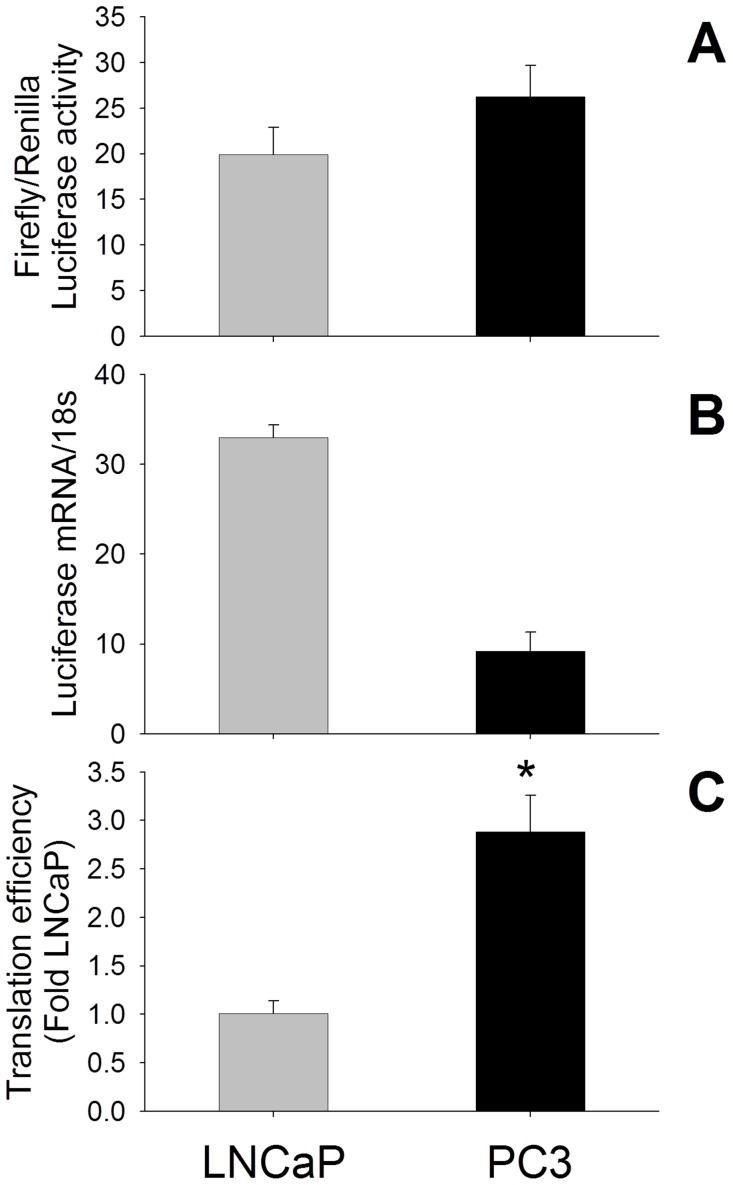
The translation efficiency of the HIF1α 5′UTR-luciferase reporter in prostate cancer cells. (A) *Firefly* and *Renilla* luciferase activities in prostate cancer cells following transfection of a HIF1α 5′UTR-luciferase construct and the pTK-Renilla control reporter vector were determined using a dual luciferase assay. (B) Real-time PCR (RT-PCR) analysis of luciferase mRNA in PC cells transfected with the HIF1α 5′UTR-luciferase construct. Following transfection, RNA was isolated, and luciferase mRNA expression detected by real time RT-PCR and normalized by 18S mRNA expression. (C) Translational efficiency represents the ratio of *Firefly*/*Renilla* luciferase activity, divided by the relative luciferase mRNA concentration in PC cells. The translational efficiency of luciferase mRNA driven by the 5′UTR region of HIF1α in PC3 cells is higher than in LNCaP cells. Values are the mean ± SEM of at least three separate experiments. *, P<0.05 versus treated LNCaP cells.

Although the regulation of translation by the 5′UTR is a major mechanism for post-transcriptional regulation of gene expression, the translation efficiency of HIF1α mRNA in prostate cells has not been reported previously. As shown in Figure 3A the ratio of *Firefly* to *Renilla* luciferase activity following the transfection of HIF1α 5′UTR-LUC reporter and pTK-Renilla control vectors was 20±3-fold and 26±3-fold in LNCaP and PC3 cells, respectively, compared to cells transfected with the empty pGL4 vector. However luciferase mRNA expression was 33±1.4-fold in LNCaP and 9±2.1-fold in PC3 cells compared to empty pGL4 vector-transfected cells (Figure 3B). Further the translation efficiency of HIF1α mRNA in PC3 cells was 2.9±0.4-fold higher compared to LNCaP cells when evaluated using the index of luciferase activity/relative mRNA content (Figure 3C).

### HIF1α Protein Expression in Human Prostate Cancer Tumors

**Figure 4:**
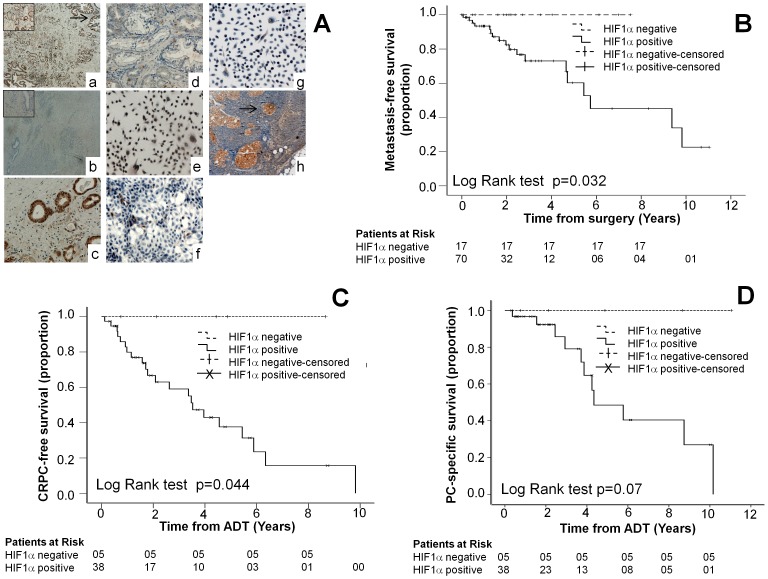
Kaplan–Meier Estimates of CRPC-free survival, metastasis-free survival and prostate cancer specific survival in patients. (A) Representative immunohistochemistry results showing expression of HIF1α in prostate cancer specimens and cell lines. Positive (Aa) and negative (Ab) staining for HIF1α was observed in two typical tumors with Gleason score 9 (Inset box, X20 view). Positive (Ac) and negative (Ad) staining was also observed in two typical tumors with Gleason score 6. Positive staining in PC3 cells (Ae) and negative staining in LNCaP cells (Af) and in HIF1α knockdown PC3 cells (Ag) demonstrated the specificity of the HIF1α antibody. Additionally, HIF1α was expressed throughout the tumor with increased expression in the main prostatic glands (indicated by the arrow in Aa) and in lymph node metastases (indicated by the arrow in Ah). (B) The Kaplan–Meier survival curve demonstrates the metastasis–free survival versus the time from surgery. (C) The Kaplan–Meier survival curve demonstrates the CRPC- free survival versus the time from the start of androgen deprivation therapy. (D) The Kaplan–Meier survival curve demonstrates the prostate cancer specific survival versus the time from the start of androgen deprivation therapy. None of the HIF1α negative patients had any of the adverse outcomes in (B), (C) or (D). Outcome was analyzed by Log Rank (Mantel – Cox) tests.

One hundred human PC specimens were divided into two groups according to their Gleason score (≤7 (38) and >7 (62), Table 1) and HIF1α status. The expression of HIF1α was assessed by immunohistochemistry (Figure 4A). Figure 4A (a) shows a positive, and Figure 4A (b) shows a negative, HIF1α staining in two typical tumors with Gleason score 9 (Inset box, X20 view). Figure 4A (c) demonstrates a positive, and Figure 4A (d) demonstrates a negative (d), HIF1α staining in two typical tumors with Gleason score 6. Positive staining in PC3 cells (Figure 4A (e)) and negative staining in LNCaP cells (Figure 4A (f)) and in HIF1α knockdown PC3 cells (Figure 4A (g)) demonstrated the specificity of the HIF1α antibody. Additionally, HIF1α was expressed throughout the tumor with increased expression in the main prostatic glands (indicated by the arrow in Figure 4A (a)) and in lymph node metastases (indicated by the arrow in Figure 4A (h)). In positive specimens HIF1α expression was homogeneous throughout the tumor area.

**Table 1:** Patient characteristics for the groups with Gleason score ≤7 or >7 and differences in HIF1α expression between the groups.

	HIF1α positive		HIF1α negative	
	Gleason score ≤7	Gleason score >7	Gleason score ≤7	Gleason score >7
Number of patients	29	54	9	8
Mean age (years)	59.9	70.7	62.2	68.1
Tumor Stage (T1–T2)	23	9†	8	3†
Tumor Stage (T3–T4¥)	6	29†	1	4†
Median pre-interventional PSA*	6.4 (1.4 – 43)	14 (0.7 – 483.7)	5.4 (4 – 10.2)	14.8 (2 – 40.2)
Median follow-up from diagnosisin years	2.3	3.6	2.4	3.3
Patients started on androgendeprivation therapy¥	0	38	0	5

The group with Gleason score ≤7 was comprised of Gleason score <6 (6), 6 (28) and 7 (4) and the group with Gleason score >7 was comprised of scores 8 (8), 9 (52) and 10 (2).

† There were 17 patients with missing data for tumor stage in the group with Gleason score >7 and 16 of these patients were HIF1α positive.

¥ There was no significant association with HIF1α expression and Gleason scores when analyzed using Pearson’s Chi-squared test or Fisher’s exact test using two-by-two tables.

* Pre-interventional PSA was defined as PSA immediately prior to obtaining the tissue sample.

HIF1α positivity rates (76% and 87%) were similar between the Gleason groups (Table 1). Despite finding higher numbers of T3-T4 stage tumors that expressed HIF1α (78%) compared with T1-T2 stage tumors (21%), there was no significant association between HIF1α positivity and higher stage (T3-T4) tumors or Gleason scores (Table 1). 43 patients with tumors with Gleason score >7 were started on ADT. Notably, of the 23 patients who progressed to CRPC, 7 patients who were treated with chemotherapy developed resistance and all these patients were HIF1α positive.

### HIF1α as a Predictor for Progression to Metastatic PC, Development of CRPC, and Prostate Cancer-specific Death in Patients Who Commenced ADT

Metastasis-free survival was significantly decreased in patients whose tumors expressed HIF1α. CRPC-free survival was also significantly decreased in patients who were started on ADT as demonstrated by Kaplan-Meier curves and Log Rank test. Although prostate cancer-specific survival was also decreased, the differences were not statistically significant (Figure 4).

The tumors of 27 patients who progressed to metastases out of the total of 87 patients, and the tumors of 23 patients who developed CRPC out of the total of 43 patients on ADT, expressed HIF1α (Table 2). HIF1α and Gleason score were independent risk factors for development of metastases on a Cox regression analysis. Although all patients who developed CRPC while on ADT had higher Gleason scores, the expression of HIF1α was an independent risk factor for developing castrate resistance as determined by Univariate and Multivariate Cox regression analysis (Table 2). Furthermore, patients with tumors that were positive for HIF1α were at a 10-fold higher risk of developing castrate resistance (p = 0.021) and 9.8-fold higher risk of progressing to metastatic prostate cancer (p = 0.017) than patients not expressing HIF1α. Although patients whose tumors expressed HIF1α had a higher risk of PC-specific death, this difference was not statistically significant possibly due to the small sample size. In addition, 4 samples of lymph node metastatic tissue and 2 samples of distant metastatic tissue (1 bony metastasis and 1 lung metastasis) expressed HIF1α. Examination of the lymph node tissue (Figure 4A (h)) revealed that HIF1α was expressed in the tumor deposits, further demonstrating that the tumors cells over-expressing HIF1α metastasize in agreement with our *in vitro* data.

**Table 2:** Univariate and Multivariate Cox Regression analysis of the development of metastatic PC from the time of surgery and CRPC, prostate cancer specific death after starting androgen deprivation therapy.

	No. of patients with event	Univariate analysis		Multivariate analysis	
		Relative Risk (95% CI)	P value	Relative Risk (95% CI)	P value
**Progression to metastatic PC (n = 87** a **)**	**27**				
HIF1α status					
HIF1α negative	0	1.0‡		1.0‡	
HIF1α positive	27	10.7 (4.5–∞)	0.011	9.8 (3.9–∞)	0.017
Gleason score †					
Gleason ≤7	1	1.0‡		1.0‡	
Gleason >7	26	6.9 (5.8–∞)	0.002	10.7 (9.7–∞)	0.001
Age		1.1(1.0–1.1)	0.013	1.0 (1.0–1.1)	0.17
Pre-interventional PSA*		1.0 (0.99–1.00)		1.0 (1.0–1.0)	0.66
**Development of CRPC in patients on ADT (n = 43)**	**23**				
HIF1α status †					
HIF1α negative	0	1.0‡		1.0‡	
HIF1α positive	23	8.3 (3.1–∞)	0.03	10.0 (4.0–∞)	0.021
Age		1.0 (1.0–1.1)	0.404	1.0 (0.9–1.0)	0.448
Pre-interventional PSA*		1.0 (1.0–1.0)	0.349	1.0 (1.0–1.0)	0.836
**PC specific deaths in patients on ADT (n = 43)**	**13**				
HIF1α status †					
HIF1α negative	0	1.0‡		1.0‡	
HIF1α positive	13	5.7 (2.1–∞)	0.111	3.07 (1.5–∞)	0.36
Age		1.1 (1.0–1.1)	0.014	1.1(1.0–1.2)	0.38
Pre-interventional PSA*		1.0 (0.9–1.0)	0.270	1.0 (1.0–1.0)	0.78

a 13 patients excluded due to incomplete metastasis related data.

† Cox regression with Firth’s penalized maximum likelihood method. CI denotes confidence interval.

‡ This group served as the reference group in the Cox regression analysis.

* Pre-interventional PSA was defined as PSA immediately prior to obtaining the tissue sample.

### Diagnostic Accuracy of HIF1α

Given the high risk of progression to metastases and of development of CRPC and prostate cancer-specific death, the diagnostic accuracy of HIF1α in determining these outcomes was analyzed. The absence of HIF1α was highly sensitive (all 100%) with very good positive predictive values (all 100%) for a favorable prognosis in the 3 outcomes. These observations suggest that the absence of HIF1α may have potential value as a predictor of men who are unlikely to progress to metastases and develop CRPC.

## Discussion

The data presented herein indicate that HIF1α is an independent risk factor for the development of CRPC. HIF1α expression was independent of Gleason score, tumor stage or type of treatment received. The multivariate analysis revealed that the risk of developing CRPC in the ADT-treated patients whose tumors expressed HIF1α increased by 10-fold. This observation clearly indicates the importance of assessing HIF1α status, which can be used to predict those patients who will develop CRPC and/or be candidates for early use of emerging second-line hormonal therapies.

The significance of the expression of HIF1α in prostate tumors has been investigated previously. Vergis and co-workers recently reported a significant association between cytoplasmic HIF1α levels in prostate tumors and time to biochemical recurrence in a cohort of prostate cancer patients treated with radiotherapy or radical prostatectomy [27], while Yasuda and co-workers demonstrated that HIF1α expression increases relative risk of recurrence of prostate adenocarcinoma [31]. However, some studies have failed to demonstrate a significant correlation between accumulation of nuclear HIF1α in prostate tumors and PSA recurrence [32], [25]. In one such study, HIF1α expression showed no correlation with the therapeutic effects of neo-adjuvant hormone therapy post radical prostatectomy in prostate adenocarcinoma, although it was hypothesized that HIF1α might be a useful biomarker for predicting early castration resistance with hormone therapy [31]. That hypothesis has now been verified in our study. Although Gravdal and co-workers had demonstrated that castration-resistant cancers have increased HIF1α expression [26], to our knowledge no previous studies have looked at the correlation between normoxic expression of HIF1α and development of CRPC. Dai and co-workers have demonstrated that hypoxia-mediated HIF1α expression increases invasiveness in PC cell lines [16]. However, the effects of normoxic HIF1α expression in PC cells had not been investigated prior to our study. Our results demonstrate that normoxic expression of HIF1α results in high invasion and chemo-resistance similar to hypoxia-induced expression of HIF1α,

Furthermore, our study suggests the possible use of HIF1α expression as a better screening tool for the development of CRPC than other biomarkers of CRPC such as chromogranin A (CgA) [3] or circulating tumor cells [33], because of its high sensitivity and negative predictive value. Although no HIF1α expression was seen in normal prostatic tissue, the HIF1α protein expression seen in benign prostatic hyperplasia (BPH) and prostatic intra-epithelial neoplasia (PIN) results in poor specificity [24], [18]. HIF1α expression in our study was analyzed in tissue from TURPs as well as radical prostatectomy tissue, and HIF1α expression analysis can also be performed on needle prostate biopsies [31]. Therefore we are currently performing a prospective study to evaluate the utility of measurement of HIF1α expression in predicting the development of CRPC.

The data presented herein also confirm that Gleason score is an independent risk factor for developing CRPC. Although tumors with higher Gleason score were associated with a shorter time to development of CRPC among patients with metastatic disease [34], a greater Gleason score was not consistently associated with decreased survival in patients with metastatic disease starting ADT [35], [36]. This effect is also seen in our study where the HIF1α status is a better predictor of CRPC than Gleason score.

One of the possible mechanisms of castration resistance is the activation of alternate growth-promoting pathways (including those stimulated by IGF-1, EGF and HER2), which can drive the growth of PC tumors independently of androgens [37]. Increased HIF1α may facilitate the activation of alternate growth factor pathways that circumvent therapeutic attempts to control the growth of PC tumors by androgen ablation. Furthermore, the observation that HIF1α can increase AR transactivation and activate the AR signaling pathway [38] raises the possibility that HIF1α-mediated AR signaling may be another mechanism by which CRPCs grow in a low androgen environment. Given the good outcomes in HIF1α negative tumors, HIF1α expression may therefore be a characteristic phenotype of aggressive CRPC and hence HIF1α may be a molecular target for treatment of CRPC.

CRPCs are chemo-resistant, with poor response to docetaxcel [39]. Our *in vitro* results confirmed the chemo-resistant effects of HIF1α in CRPC, as knockdown of HIF1α in PC3 cells increased the sensitivity to cytotoxic treatments including oxidative stress and 5-fluorouracil. In contrast, overexpression of HIF1α in LNCaP cells increased the chemo-resistance to cytotoxic treatments. Our results are consistent with previous reports that high levels of HIF1α reduced the effectiveness of cytotoxic agents in lung and gastric cancer cell lines [40], [41], [42]. Some hypothetical mechanisms for chemo-resistance include inhibition of drug efflux, autophagy, DNA repair, and apoptosis influenced by genes directly or indirectly regulated by HIF1α [42], [43]. However further studies are required to elucidate the mechanism of HIF1α-dependent chemo-resistance in CRPC-like cells and tumors.

Nearly a decade ago, Zhong and co-workers discovered that growth factor-stimulated activity of the phosphatidylinositol 3-kinase (PI3K) pathway is responsible for the oxygen-independent constitutive overexpression of the HIF1α protein in PC3 cells [14], [23]. Although several different hypothesis have been raised to explain the overexpression of HIF1α in PC tumors and cell lines, including gene amplification [44], increased transcription of HIF1α mRNA [45], single nucleotide polymorphisms [46], expression of truncated HIF1α isoforms [19], and tumor hypoxia-dependent stabilization of HIF1α [29], there is no definitive consensus on the mechanism involved. Previously HER2 signaling in non-hypoxic MCF-7 breast cancer cells has been shown not only to affect HIF1α stability but also to increase dramatically the rate of HIF1α protein synthesis [47]. Although HIF1α signaling is upregulated in castration-resistant LNCaP C4-2 cells as compared to the parental LNCaP cells [17] no previous studies have compared the translation of HIF1α in androgen-sensitive LNCaP and androgen-insensitive PC3 cells.

In the present study we therefore investigated the hypothesis that increased translation of HIF1α mRNA is responsible for the overexpression of HIF1α protein in CRPC-like cells. Transcription of luciferase mRNA is 3-fold greater in LNCaP compared to PC3 cells but, after correction for the transfection efficiency of the HIF1α-Luc reporter construct, our results demonstrate for the first time that there is a 3-fold increase in the translational efficiency of HIF1α mRNA in PC3 cells compared to LNCaP cells. The alternative explanation that the luciferase protein is 3-fold less stable in LNCaP cells is less likely as the use of luciferase as a reporter is based on the fact that its half life is similar in a range of mammalian cells (t_½_ ∼ 3–4 hours, Promega). Mutation of the GC rich region in the HIF1α promoter sequence has previously been shown to decrease luciferase activity driven by the HIF1α promoter [48], [49]. Interestingly we have noted that this GC-rich region forms part of the 5′UTR and not the promoter due to a shift in the newly annotated transcription start site in the most recent sequence of HIF1α mRNA in PubMed (NCBI Reference Sequence: NM_001530.3) compared to the previously identified transcription start site of the *HIF1α* gene [50]. The possibility that the decrease in the luciferase activity observed by De Armond and co-workers [49] may be due to a decrease in the translation rather than transcription of luciferase mRNA deserves further attention. Additional studies are also warranted to determine the role of the GC-rich region and 5′UTR in the increased translation of HIF1α in prostate cancer cells, especially when enhanced protein synthesis from a specific subset of mRNAs that contain highly structured (GC-rich) 5′UTRs is one of the hallmarks of cancer [51], [52], [53]. In this context it is pertinent to note that a G-rich oligonucleotide has been shown to inhibit HIF1α expression in PC3 cells [54].

Previously hypoxia-induced HIF1α had been shown to increase migration and chemo-resistance in PC cells [16]. In the current study we have shown that normoxic HIF1α also increases metastatic potential and chemo-resistance in PC cells and HIF1α expressing human prostate tumors have poor outcomes. Although expression of HIF1α was heterogeneous in tumor sections it would be difficult in the current study to predict whether up regulation of HIF1α is hypoxia-dependent or -independent (normoxic). The current paradigm is that tumor hypoxia increases HIF1α expression in prostate cancer. Interestingly the expression of HIF1α can also be increased under a normoxic environment at all three levels of regulation (transcription, translation and protein stability [55]) and overexpression of HIF1α under normoxia has been detected in various cancers [56], [57], [58], [59]. Unlike the case of melanoma where an increase in HIF1α expression under normoxia is due to increased HIF1α protein stability [59], we have shown that in PC cells the increase in HIF1α may be due to increased HIF1α protein translation. The amount of HIF1α mRNA in the metastatic advanced PC tumors is no different from that in normal tissue [45] and therefore the possibility that in more advanced metastatic CRPC tumors HIF1α expression is increased via a translational mechanism rather a post-translational (oxygen-dependent stability) or transcriptional pathway needs to be investigated. Regardless of the hypoxic condition of the tumor, therapeutic inhibition of HIF1α may be of use in the treatment of metastatic prostate cancer. Furthermore a precise understanding of the mechanisms behind the increased translation of HIF1α protein in PC cells may not only lead to identification of novel therapeutic targets to inhibit HIF1α expression, but may also guide the choice of which HIF1α inhibitor from the many available may work best in CRPC patients [60], [61].

Recently the cardiac glycoside digoxin has been shown to inhibit HIF1α mRNA translation and PC3 tumor xenograft growth in mice [62]. In a large prospective cohort study, Platz and co-workers have demonstrated that men who used digoxin had a 25% lower risk of prostate cancer, including disease that was potentially lethal [63]. A plausible explanation for these observations is that CRPC patients whose tumors express HIF1α are most likely to respond favorably to digoxin in combination with chemotherapy.

In conclusion HIF1α-positive tumors have a worse prognosis compared to HIF1α-negative tumors. HIF1α expression is a better indicator of PC-specific survival than Gleason score alone, and could therefore be used to predict castrate resistance and hence prognosis in tumors with high Gleason score. HIF1α expression in CRPCs possibly contributes to chemo-resistance and tumor metastasis. Although HIF1α inhibitors are currently being evaluated in clinical trials for treatment of various tumors [13], to our knowledge no trial is being conducted in CRPC. The combination of HIF inhibitors with cytotoxic agents would seem worthy of testing in CRPC. Targeted reduction of HIF1α may increase the responsiveness of CRPCs to chemotherapy and thus lead to better clinical prognosis and survival.

## Acknowledgments

We would like to thank Carmel Murone and Dr. Lee Lewis of the Victorian Cancer Biobank for their tireless efforts in data and tissue supply. We would also like to thank Dr. Sandy Clarke, Statistician, University of Melbourne for statistical advice and Mr. Shomik Sengupta (Urologist), Mr. Joseph Ischia (Urologist) and Mr. Raj Persad (Urologist - Bristol Royal Infirmary, UK) for their constructive comments and support.
